# Supplementary material for: A Critical View of the Application of the APEX Software (Aqueous Photochemistry of Environmentally-Occurring Xenobiotics) to Predict Photoreaction Kinetics in Surface Freshwaters
Source: Molecules. 2019 Dec 18;25(1):9. doi: 10.3390/molecules25010009 (PMC7017383; doi:10.3390/molecules25010009)
Supplement: Supplementary file 1 [file molecules-25-00009-s001.zip › 3.2.4_gcc-4.4.0.zip!/3.2.4_gcc-4.4.0/3.2.4_gcc-4.4.0/doc/octave/html/interpreter/Two_002ddimensional-Function-Plotting.html]

Two-dimensional Function Plotting - Untitled


Up: Two-Dimensional Plots


---

##### 15.1.1.1 Two-dimensional Function Plotting

Octave can plot a function from a function handle inline function or
string defining the function without the user needing to explicitly
create the data to be plotted. The function `fplot` also generates
two-dimensional plots with linear axes using a function name and limits
for the range of the x-coordinate instead of the x and y data. For
example,

```
     fplot (@sin, [-10, 10], 201);
```

produces a plot that is equivalent to the one above, but also includes a
legend displaying the name of the plotted function.

— Function File: **fplot** (fn, limits)  
— Function File: **fplot** (fn, limits, tol)  
— Function File: **fplot** (fn, limits, n)  
— Function File: **fplot** (..., fmt)  
> Plot a function fn, within the defined limits. fn
> an be either a string, a function handle or an inline function.
> The limits of the plot are given by limits of the form
> `[`xlo`,` xhi`]` or `[`xlo`,` xhi`,` ylo`,` yhi`]`. tol is the default tolerance to use for the
> plot, and if tol is an integer it is assumed that it defines the
> number points to use in the plot. The fmt argument is passed
> to the plot command.
>
> ```
>              fplot ("cos", [0, 2*pi])
>              fplot ("[cos(x), sin(x)]", [0, 2*pi])
> ```
>
>
>
>
> **See also:** plot.

Other functions that can create two-dimensional plots directly from a
function include `ezplot`, `ezcontour`, `ezcontourf` and
`ezpolar`.

— Function File: **ezplot** (f)  
— Function File: **ezplot** (fx, fy)  
— Function File: **ezplot** (..., dom)  
— Function File: **ezplot** (..., n)  
— Function File: **ezplot** (h, ...)  
— Function File: h = **ezplot** (...)  
> Plots in two-dimensions the curve defined by f. The function
> f may be a string, inline function or function handle and can
> have either one or two variables. If f has one variable, then
> the function is plotted over the domain `-2*pi <` x `< 2*pi`
> with 500 points.
>
> If f has two variables then f`(`x`,`y`) = 0`
> is calculated over the meshed domain `-2*pi <` x `|` y `< 2*pi` with 60 by 60 in the mesh. For example
>
> ```
>           ezplot (@(x, y) x .^ 2 - y .^ 2 - 1)
> ```
>
> If two functions are passed as strings, inline functions or function
> handles, then the parametric function
>
> ```
>           x = fx (t)
>           y = fy (t)
> ```
>
> is plotted over the domain `-2*pi <` t `< 2*pi` with 500
> points.
>
> If dom is a two element vector, it represents the minimum and maximum
> value of x, y and t. If it is a four element
> vector, then the minimum and maximum values of x and t
> are determined by the first two elements and the minimum and maximum
> of y by the second pair of elements.
>
> n is a scalar defining the number of points to use in plotting
> the function.
>
> The optional return value h provides a list of handles to the
> the line objects plotted.
>
>
>
> **See also:** plot, ezplot3.

— Function File: **ezcontour** (f)  
— Function File: **ezcontour** (..., dom)  
— Function File: **ezcontour** (..., n)  
— Function File: **ezcontour** (h, ...)  
— Function File: h = **ezcontour** (...)  
> Plots the contour lines of a function. f is a string, inline function
> or function handle with two arguments defining the function. By default the
> plot is over the domain `-2*pi <` x `< 2*pi` and `-2*pi <` y `< 2*pi` with 60 points in each dimension.
>
> If dom is a two element vector, it represents the minimum and maximum
> value of both x and y. If dom is a four element vector,
> then the minimum and maximum value of x and y are specify
> separately.
>
> n is a scalar defining the number of points to use in each dimension.
>
> The optional return value h provides a list of handles to the
> the parts of the vector field (body, arrow and marker).
>
> ```
>           f = @(x,y) sqrt(abs(x .* y)) ./ (1 + x.^2 + y.^2);
>           ezcontour (f, [-3, 3]);
> ```
>
>
>
>
> **See also:** ezplot, ezcontourf, ezsurfc, ezmeshc.

— Function File: **ezcontourf** (f)  
— Function File: **ezcontourf** (..., dom)  
— Function File: **ezcontourf** (..., n)  
— Function File: **ezcontourf** (h, ...)  
— Function File: h = **ezcontourf** (...)  
> Plots the filled contour lines of a function. f is a string, inline
> function or function handle with two arguments defining the function. By
> default the plot is over the domain `-2*pi <` x `< 2*pi` and
> `-2*pi <` y `< 2*pi` with 60 points in each dimension.
>
> If dom is a two element vector, it represents the minimum and maximum
> value of both x and y. If dom is a four element vector,
> then the minimum and maximum value of x and y are specify
> separately.
>
> n is a scalar defining the number of points to use in each dimension.
>
> The optional return value h provides a list of handles to the
> the parts of the vector field (body, arrow and marker).
>
> ```
>           f = @(x,y) sqrt(abs(x .* y)) ./ (1 + x.^2 + y.^2);
>           ezcontourf (f, [-3, 3]);
> ```
>
>
>
>
> **See also:** ezplot, ezcontour, ezsurfc, ezmeshc.

— Function File: **ezpolar** (f)  
— Function File: **ezpolar** (..., dom)  
— Function File: **ezpolar** (..., n)  
— Function File: **ezpolar** (h, ...)  
— Function File: h = **ezpolar** (...)  
> Plots in polar plot defined by a function. The function f is either
> a string, inline function or function handle with one arguments defining
> the function. By default the plot is over the domain `0 <` x `<
> 2*pi` with 60 points.
>
> If dom is a two element vector, it represents the minimum and maximum
> value of both t. n is a scalar defining the number of points to
> use.
>
> The optional return value h provides a list of handles to the
> the parts of the vector field (body, arrow and marker).
>
> ```
>           ezpolar (@(t) 1 + sin (t));
> ```
>
>
>
>
> **See also:** polar, ezplot, ezsurf, ezmesh.
